# Serum Procalcitonin Levels in Acute Encephalopathy with Biphasic Seizures and Late Reduced Diffusion

**DOI:** 10.1155/2018/2380179

**Published:** 2018-03-14

**Authors:** Yosuke Fujii, Masato Yashiro, Mutsuko Yamada, Tomonobu Kikkawa, Nobuyuki Nosaka, Yukie Saito, Kohei Tsukahara, Masanori Ikeda, Tsuneo Morishima, Hirokazu Tsukahara

**Affiliations:** ^1^Department of Pediatric Acute Medicine, Okayama University Graduate School of Medicine, Dentistry and Pharmaceutical Sciences, Okayama, Japan; ^2^Department of Pediatrics, Okayama University Graduate School of Medicine, Dentistry and Pharmaceutical Sciences, Okayama, Japan; ^3^Department of Emergency and Critical Care Medicine, Okayama University Graduate School of Medicine, Dentistry and Pharmaceutical Sciences, Okayama, Japan

## Abstract

Procalcitonin (PCT) is used as a biomarker in severe infections. Here, we retrospectively investigated levels of serum PCT, C-reactive protein (CRP), and inflammatory cytokines (IL-6, TNF-*α*, and IFN-*γ*) in the second phase of patients with acute encephalopathy with biphasic seizures and late reduced diffusion (AESD). Nine AESD pediatric patients (4 men, 5 women; AESD group) admitted to Okayama University Hospital from 2010 to 2016 were compared with 10 control patients with febrile seizures (FS) (3 men, 7 women; FS group). Mean PCT concentrations (ng/mL) in the AESD and FS groups were significantly different, at 9.8 ± 6.7 and 0.8 ± 0.9, respectively (*p* = 0.0006). CRP (mg/dL) were 0.79 ± 0.89 and 1.4 ± 1.0 (*p* = 0.94), respectively; IL-6 (pg/mL) were 449.7 ± 705.0 and 118.3 ± 145.4 (*p* = 0.20), respectively; TNF-*α* (pg/mL) were 18.6 ± 12.5 and 16.6 ± 6.0 (*p* = 0.67), respectively; and IFN-*γ* (pg/mL) were 79.6 ± 158.5 and 41.9 ± 63.7 (*p* = 0.56), respectively. Ratios of PCT to CRP were 27.5 ± 34.2 and 3.2 ± 6.8 (*p* < 0.0001), respectively. The sensitivity and specificity in the diagnosis of AESD using a cutoff of PCT/CRP ratio of 1.0 were 79% and 100%, respectively. These results suggest that PCT and the PCT/CRP ratio are useful in auxiliary diagnosis of the second stage of AESD, and in AESD, PCT is likely to increase through a different mechanism.

## 1. Introduction

Procalcitonin (PCT) is a biomarker of systemic infection. It is a more specific marker of microbial infections than C-reactive protein (CRP) and is used in the diagnosis and assessment of therapeutic effect in sepsis and other systemic infections [1]. Acute encephalopathy (AE) is a severe complication causing brain dysfunction by a viral infection such as influenza virus or human herpesvirus 6 (HHV-6). AE with biphasic seizures and late reduced diffusion (AESD) is one of the clinical subtypes of AE, which is characterized by a febrile seizure (usually >30 min) as the initial neurological symptom, followed by secondary seizures at days 4 to 6 [2]. Here, we retrospectively investigated levels of serum PCT, CRP, and inflammatory cytokines (IL-6, TNF-*α*, and IFN-*γ*) in patients with AESD.

## 2. Materials and Methods

Nine AESD patients (4 men, 5 women; AESD group) admitted to the Department of Pediatrics or the Department of Emergency and Critical Care Medicine, Okayama University Hospital, during the 7-year period from 2010 to 2016 were compared with 10 control patients with complex febrile seizures (3 men, 7 women; FS group). The AESD criteria used were the characteristic clinical course which had secondary seizures within a week after febrile seizures, electroencephalogram (EEG) abnormalities characterized by generalized high voltage slow waves and high signal foci in the subcortical white matter on diffusion-weighted head MR images taken at the start of therapy or during follow-up. The criteria of the FS group were status epilepticus lasting ≥15 min plus a clinical course eliminating the diagnosis of encephalopathy.

Serum PCT, CRP, and cytokines were measured at the second phase of AESD (before treatment) and onset of complex febrile seizures. In AESD, the changes in PCT and CRP levels during the follow-up period were measured. The measured cytokines were IL-6, TNF-*α*, and IFN-*γ*, using a Human Cytokine/Chemokine-Magnetic Bead Panel (Merck Millipore, Germany) with a Luminex 100 system (Merck Millipore).

Statistical analysis was performed using Prism 6.0 software. Wilcoxon's signed-rank test was used to evaluate the statistical significance of differences between the two groups (*p* < 0.05).

This study was approved by the Ethics Committee of the Okayama University Graduate School of Medicine, Dentistry and Pharmaceutical Sciences and Okayama University Hospital (approval number: 1706-033).

## 3. Results

### 3.1. Patient Characteristics (Table 1)

The average period from the first seizure to the second seizure in the AESD group was 5.3 days (range 4–7). Mean age at AESD onset was 13.9 (range 10–29) months, and pathogens of the preceding infection were influenza A virus (2 cases) and HHV-6 (7 cases). All blood cultures were negative in the AESD group. Mean age of FS onset was 26.6 (range 11–44) months, and causative agents were HHV-6 (2 cases), adenovirus (1 case), human metapneumovirus (1 case), hemolytic streptococcus (1 case), and unknown (6 cases).

All patients were treated in accordance with the guidelines for the management of influenza-associated encephalopathy from the time they were diagnosed as AESD. They received pulse steroid therapy, immunoglobulin therapy, mannitol, and edaravone, and five of them received targeted temperature management [3].

### 3.2. PCT, CRP, and Cytokine Levels at the Start of Therapy (Table 2)

Mean PCT concentrations were significantly higher in the AESD group than in the FS group (9.8 ± 6.7 ng/mL and 0.8 ± 0.9 ng/mL, respectively; *p* = 0.0006), and 7 out of 9 patients had PCT levels exceeding the cutoff for severe sepsis (2 ng/mL). Similarly, CRP concentrations were 0.79 ± 0.89 mg/dL and 1.4 ± 1.0 mg/dL (*p* = 0.94), respectively; mean IL-6 concentrations were 449.7 ± 705.0 pg/mL and 118.3 ± 145.4 pg/mL (*p* = 0.20), respectively; TNF-*α* concentrations were 18.6 ± 12.5 pg/mL and 16.6 ± 6.0 pg/mL (*p* = 0.67), respectively; and IFN-*γ* concentrations were 79.6 ± 158.5 pg/mL and 41.9 ± 63.7 pg/mL (*p* = 0.56), respectively.

**Figure 1:**
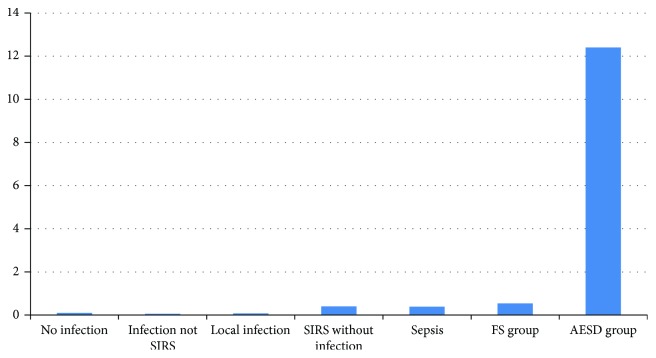
AESD: acute encephalopathy with biphasic seizures and late reduced diffusion; CRP: C-reactive protein; FS: febrile seizures; PCT: procalcitonin; SIRS: systemic inflammatory response syndrome. PCT/CRP ratio = PCT average level (ng/mL)/CRP average level (mg/dL). Data for the FS and AESD groups are from this study; other data is from Nakanishi et al. [4].

**Figure 2:**
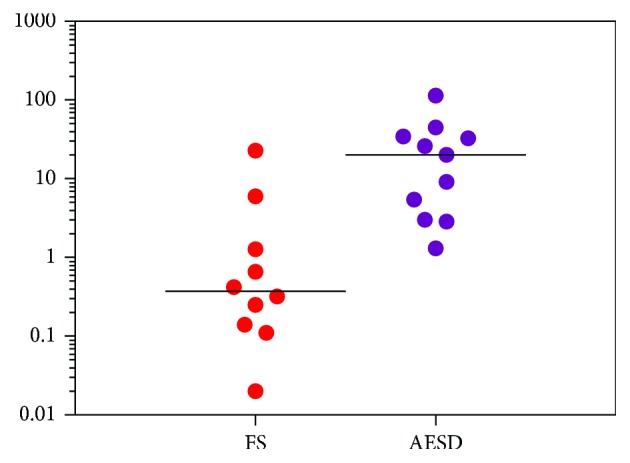
AESD: acute encephalopathy with biphasic seizures and late reduced diffusion; CRP: C-reactive protein; FS: febrile seizures; PCT: procalcitonin. PCT/CRP ratio = PCT level (ng/mL)/CRP level (mg/dL).

Elevation of CRP was less prominent compared with that of PCT in AESD, resulting in a significantly higher PCT/CRP ratio in the AESD group (27.5 ± 34.2) than in the FS group (3.2 ± 6.8) (*p* < 0.0001). CRP levels often increase with elevation of PCT levels in sepsis [4, 5].  Figure 1 shows a comparison of PCT/CRP ratios in patients with AESD and in those with other diseases reported in this study or a previous study [4]. Ratios of all other diseases were lower than 1.0. All patients had a PCT/CRP ratio > 1.0 in the AESD group, compared with only 3 out of 10 in the FS group (Figure 2). The diagnosis of AESD using a cutoff of a PCT/CRP ratio of 1.0 gave a sensitivity of 79% and a specificity of 100%.

### 3.3. Time-Course Changes in PCT and CRP Levels (Figure 3)

PCT and CRP levels were measured on consecutive days during follow-up in 5 AESD patients. In this figure, the day of the secondary seizure is defined as day 0. PCT peaked in the very early stage of therapy (0-1 days after the start of therapy); however, the peak of CRP was delayed (0–3 days after the start of therapy).

## 4. Discussion

There are three main categories in AE. The first group typically presents with cytokine storm and cerebral edema, the second with AE with status epilepticus, and the third with AE caused by metabolic derangement. The second group AESD has become the most common (29%) in recent years [6]. The pathogenic mechanism is hypothesized excitotoxic injury with delayed neuronal death [2]. Although the fatality is low (1.4%), AESD is difficult to diagnose, particularly in the early stage, and sequelae occur in a substantial proportion of AESD patients (66.2%) [6]. Thus, identifying markers for early diagnosis and developing effective therapies for AESD is eagerly awaited [7].

PCT is a 13 kDa protein usually produced in the thyroid as a precursor of calcitonin, a hormone involved in calcium metabolism. However, in severe infections, it is produced in the lungs and liver upon induction by endotoxins, IL-6, and/or TNF-*α*. IFN-*γ* inhibits the synthesis of TNF-*α*, thereby suppressing PCT production. Generally, sepsis and severe sepsis are suspected when serum PCT levels exceed 0.5 and 2.0 ng/mL, respectively [8]. However, it increases in systemic fungal infections, acute respiratory distress syndrome, acute pancreatitis, and Kawasaki disease, and thus, PCT levels need to be judged carefully [9].

In the present study, we found several AESD patients with significant increase of PCT in the second phase. In reports on the relationship between AE and PCT, Takasu et al. report that in a retrospective study of 7 cases of AESD and 8 cases of febrile status epilepticus, PCT on the first day was significantly higher in the AESD group [10], and Azuma et al. report that in a retrospective study of 7 cases of AESD, mean PCT concentrations were 16.2 pg/mL and 3 cases had PCT rising before MRI finding [11].

The present retrospective study comparing PCT levels between the AESD and FS groups revealed that PCT was significantly elevated in the second phase of AESD. Also, elevation of CRP was less prominent compared with that of PCT in AESD, resulting in a significantly higher PCT/CRP ratio in the AESD group than in the FS group. Discrepancies in concentration changes between PCT and CRP may be explained by (1) differences in cytokine kinetics and (2) differences in PCT-producing cells and in production mechanisms. Inflammatory cytokines such as IL-6 and TNF-*α* are involved in the pathology of encephalopathy characterized by cytokine storm. In AESD, levels of inflammatory cytokines are increased in the cerebrospinal fluid but only slightly in blood [12]. The present study did not find significant differences in levels of cytokines involved in PCT production, such as serum IL-6, TNF-*α*, and IFN-*γ*, between the AESD and FS groups, suggesting that their effects are negligible. In AESD, PCT is likely to increase through a different mechanism from that obtained in sepsis, such as via the involvement of distinct PCT-producing cells.

PCT level and PCT/CRP ratio appear to be useful markers in the diagnosis of AESD: patients with a PCT/CRP ratio > 1.0 are likely to have developed AESD. Future studies with more cases will help further elucidate its clinical significance and response mechanisms, which presently remain unclear. AESD is difficult to diagnose in the early stage, and brain dysfunction has often already occurred when the disease is diagnosed.

The measurement of PCT and PCT/CRP ratio are safer and easier than MRI or EEG for children, and they may lead to auxiliary diagnosis of AESD. If we can prove that PCT will rise before the second seizure attack, we can anticipate the onset of AESD and intervene at an early stage.

## 5. Conclusion

This study investigated PCT and CRP levels in the second stage of AESD. PCT level increased significantly, while CRP level did not increase in comparison. Furthermore, PCT/CRP ratio was extremely high in AESD compared with other conditions. There were no significant differences in levels of cytokines involved in PCT production, such as serum IL-6, TNF-*α*, and IFN-*γ*, between the AESD and FS groups. In AESD, PCT is likely to increase through a different mechanism from that obtained in sepsis.

## Figures and Tables

**Figure 3:**
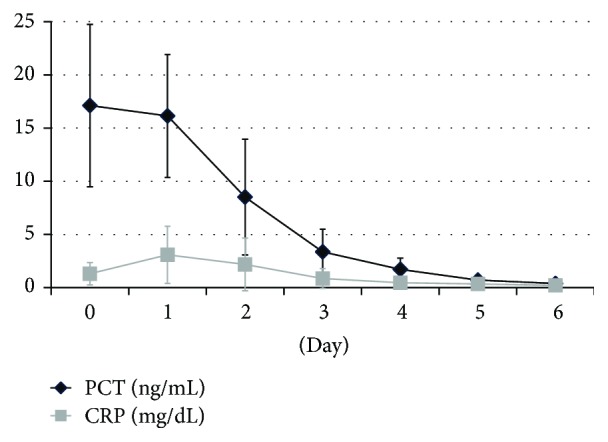
CRP: C-reactive protein; mPSL pulse: methyl prednisolone pulse therapy; PCT: procalcitonin. Follow-up after diagnosis of acute encephalopathy with biphasic seizures and late reduced diffusion and start of intervention. The day of secondary seizure is defined as day 0. Data are presented as the mean ± standard deviation.

**Table 1:** 

	AESD group (*n* = 9)	FS group (*n* = 10)
Age, months	13.9 (10–29)	26.6 (11–44)
Sex (M/F), *n*	4/5	3/7
Pathogens^∗^, *n*	HHV-6: 7 Influenza: 2	HHV-6: 2 Adenovirus: 1 hMPV: 1 Hemolytic streptococcus: 1 Unknown: 6
Sequelae, *n*	None: 5 Mild: 2 Severe: 2	None: 10

AESD: acute encephalopathy with biphasic seizures and late reduced diffusion; FS: febrile seizures; HHV-6: human herpesvirus-6; hMPV: human metapneumovirus. ^∗^All AESD specimens were subjected to blood culture, and the results were all negative.

**Table 2:** 

	AESD group	FS group	*p* value
PCT (ng/mL)	9.8 ± 6.7	0.8 ± 0.9	0.0011
CRP (mg/dL)	0.79 ± 0.89	1.4 ± 1.0	0.21
PCT/CRP^∗^	27.5 ± 34.2	3.2 ± 6.8	<0.0001
IL-6 (pg/mL)	449.7 ± 705.0	118.3 ± 145.4	0.20
TNF-*α* (pg/mL)	18.6 ± 12.5	16.6 ± 6.0	0.67
IFN-*γ* (pg/mL)	79.6 ± 158.5	41.9 ± 63.7	0.56

AESD: acute encephalopathy with biphasic seizures and late reduced diffusion; CRP: C-reactive protein; FS: febrile seizures; PCT: procalcitonin. Data are presented as the mean ± standard deviation. ^∗^The ratio was calculated using a CRP value of 0.15 when it is below 0.15.
