# Corrigendum to “Serum Procalcitonin Levels in Acute Encephalopathy with Biphasic Seizures and Late Reduced Diffusion”

**DOI:** 10.1155/2019/4025694

**Published:** 2019-12-13

**Authors:** Yosuke Fujii, Masato Yashiro, Mutsuko Yamada, Tomonobu Kikkawa, Nobuyuki Nosaka, Yukie Saito, Kohei Tsukahara, Masanori Ikeda, Tsuneo Morishima, Hirokazu Tsukahara

**Affiliations:** ^1^Department of Pediatric Acute Medicine, Okayama University Graduate School of Medicine, Dentistry and Pharmaceutical Sciences, Okayama, Japan; ^2^Department of Pediatrics, Okayama University Graduate School of Medicine, Dentistry and Pharmaceutical Sciences, Okayama, Japan; ^3^Department of Emergency and Critical Care Medicine, Okayama University Graduate School of Medicine, Dentistry and Pharmaceutical Sciences, Okayama, Japan

In the article titled “Serum Procalcitonin Levels in Acute Encephalopathy with Biphasic Seizures and Late Reduced Diffusion” [1], there are errors that should be updated as follows:

1. In Abstract, (*p* = 0 0006) should be (*p* = 0.0011), (*p* = 0 94) should be (*p* = 0.21), and (79% and 100%) should be (100% and 80%)
2. In Section 3.2 in Results, “a sensitivity of 79% and a specificity of 100%” should be “a sensitivity of 100% and specificity of 80%”
3. The figures' and tables' captions are incomplete. They should be updated as follows:

Figure 1 PCT/CRP ratio in each disease group. AESD: acute encephalopathy with biphasic seizures and late reduced diffusion; CRP: C-reactive protein; FS: febrile seizures; PCT: procalcitonin; SIRS: systemic inflammatory response syndrome. PCT/CRP ratio = PCT level (ng/mL)/CRP level (mg/dL). Data for the FS and AESD groups are from this study; other data is from Nakanishi et al.

Figure 2 PCT/CRP ratio in FS and AESD. AESD: acute encephalopathy with biphasic seizures and late reduced diffusion; CRP: C-reactive protein; FS, febrile seizures; PCT: procalcitonin. PCT/CRP ratio = PCT level (ng/mL)/CRP level (mg/dL).

Figure 3 Time course of PCT and CRP after onset in AESD. CRP: C-reactive protein; mPSL pulse: methyl prednisolone pulse therapy; PCT: procalcitonin. Follow-up after diagnosis of acute encephalopathy with biphasic seizures and late reduced diffusion and start of intervention. Data are presented as the mean ± standard deviation.

Table 1 Patient characteristics of AESD and FS. AESD: acute encephalopathy with biphasic seizures and late reduced diffusion; FS: febrile seizures; HHV-6: human herpesvirus-6; hMPV: human metapneumovirus. ^∗^All AESD specimens were subjected to blood culture, and results were all negative.

Table 2 PCT, CRP, and inflammatory cytokine of AESD and FS. AESD: acute encephalopathy with biphasic seizures and late reduced diffusion; CRP: C-reactive protein; FS: febrile seizures; PCT: procalcitonin. Data are presented as the mean ± standard deviation. ^∗^The ratio was calculated using a CRP value of 0.15 when it is below 0.15.
